# The impact of negative life events on internalizing problems in adolescents: the chain mediating effects of physical activity and emotional competence

**DOI:** 10.3389/fpsyg.2026.1854312

**Published:** 2026-08-04

**Authors:** Yaning Wang, Jiaxin Lyu, Hao Hong, Jixiang Wang, Shen Qiao

**Affiliations:** 1 School of Wushu, Henan University, Kaifeng, China; 2 Faculty of Education, Henan University, Kaifeng, China

**Keywords:** adolescents, emotional competence, internalizing problems, negative life events, physical activity

## Abstract

**Background:**

Adolescents are particularly vulnerable to developing internalizing problems (IPs) when exposed to Negative Life Events (NLEs) during their development. Physical Activity (PA) has been shown to exert beneficial effects on stress coping and Emotional Competence (EC). This study aimed to examine the association between NLEs and IPs in adolescents and to investigate the chain mediating roles of PA and EC, so as to provide a theoretical basis for the early prevention and intervention of IPs in this population.

**Methods:**

A total of 902 junior and senior high school students from Henan Province, China, were recruited for this study. Participants were assessed using the Adolescent Self-Rating Life Events Checklist, the Strengths and Difficulties Questionnaire, the Physical Activity Rating Scale, and the Emotional Competence Scale. Statistical analyses were performed using SPSS 27.0 and the PROCESS macro to test the mediation model.

**Results:**

(1) NLEs were significantly and positively correlated with IPs, but significantly and negatively correlated with PA and EC. PA was significantly and positively correlated with EC. (2) Regression analyses indicated that NLEs were positively associated with IPs, while significantly and negatively predicting PA. PA was positively associated with EC, and both PA and EC were significantly and negatively associated with IPs. (3) Mediation analyses revealed that the independent mediating effect of PA was significant (b = 0.023, 95%CI [0.012, 0.034]); the independent mediating effect of EC was also significant (b = 0.021, 95%CI [0.013, 0.031]); moreover, the chain mediating effect of PA and EC was significant (b = 0.009, 95%CI [0.006, 0.014]).

**Conclusion:**

NLEs were identified as an important correlate of IPs in adolescents. PA and EC showed potential independent and sequential indirect associations in the relationship between NLEs and IPs. These findings suggest that PA and EC may represent potential indirect mechanisms linking NLEs and IPs among adolescents. However, given the cross-sectional design, these pathways should be interpreted as theoretically informed associations rather than causal processes.

## 1 Introduction

Adolescence represents a critical stage in individual psychological development and socialization. During this period, psychological functions such as Emotional Competence (EC) and cognitive control remain in a highly sensitive and plastic phase. Because psychological structures and behavioral patterns have not yet fully stabilized, they are particularly susceptible to substantial changes under the influence of both internal and external environmental factors (Dahl et al., 2018). As one of the many stressors that may trigger mental health problems in adolescents, Negative Life Events (NLEs) can elicit intense psychological stress responses when their frequency and intensity exceed adolescents’ still-developing psychological tolerance and EC capacities (Chen et al., 2026). Under such circumstances, adolescents are more likely to develop negative attributional styles and feelings of helplessness, which may gradually evolve into internalizing problems (IPs) characterized by anxiety and depression. Epidemiological data indicate that approximately one in seven adolescents worldwide is affected by mental health problems (Kapadia, 2024; Wang et al., 2025c). In China, adolescent mental health is likewise a growing concern, and large-scale survey data have shown that the detection rates of IPs, including depression and anxiety, among junior and senior high school students have been rising year by year (Zhou et al., 2024).

As a pattern of excessive inward emotional focus and regulatory difficulty, IPs are primarily manifested in the tendency to direct distress inward, thereby giving rise to negative emotional states such as anxiety, depression, and somatic symptoms (Luijten et al., 2021). IPs often originate from early childhood tendencies toward anxiety or depression and may develop into more complex clinical internalizing disorders during adolescence, such as generalized anxiety disorder or major depressive disorder (Colder et al., 2018). Existing studies have confirmed that individuals exposed to NLEs are more likely to develop internalizing and other pathological psychological problems. Because these problems are often covert in presentation, they are easily overlooked by parents and peers and may persist over time (Geng et al., 2022). Without timely intervention, IPs can not only substantially impair adolescents’ social functioning and academic adjustment, but also increase the risk of suicide, thereby imposing a sustained burden on families and public mental health systems. Therefore, a systematic investigation of the pathways underlying adolescents’ IPs is of considerable theoretical and practical significance.

At present, under the combined pressures of modern stressors such as academic burden, social media exposure, and social injustice, adolescents’ IPs are becoming increasingly severe (Melchior and Davisse-Paturet, 2025), posing significant challenges to both youth mental health promotion and public mental health systems (Armitage et al., 2024). Notably, however, not all adolescents exposed to comparable environmental stress develop IPs, suggesting that certain protective factors may be involved in the association between external stress and internal psychological symptoms (Wang J. et al., 2024). To date, most relevant studies have focused primarily on the main effects of stress, whereas the mechanisms potentially involved in these associations remain insufficiently explored. Accordingly, the present study aims to systematically examine potential pathways linking NLEs with adolescents’ IPs by incorporating Physical Activity (PA) and EC into an integrated analytical framework, with a view to providing a theoretical basis for the early prevention and intervention of psychological problems.

### 1.1 Relationship between Negative Life Events and internalizing problems

NLEs refer to life experiences that impose significant psychological stress or distress on individuals and constitute a key risk factor in adolescent development (Low et al., 2012; Brown et al., 2020). Previous research has shown that persistent exposure to NLEs can weaken individuals’ sense of control, making adolescents more likely to develop negative attributional styles and, which may be related to higher levels of depressive symptoms (Gee et al., 2022). A limited number of studies have further confirmed the robust association between NLEs and IPs across different populations and contexts. For instance, in samples of Chinese secondary school students, daily NLEs such as academic stress and interpersonal conflict have been found to significantly and positively predict subsequent levels of depression and anxiety, with these effects persisting over time (Wang et al., 2025b). Similarly, another large-scale survey study reported that adolescents who had experienced family adversities, such as parental divorce or the death of a loved one, showed a significantly higher prevalence of internalizing symptoms than their peers in the general population (Wang et al., 2022). In addition, chronic exposure to stress may lead to dysregulation of the hypothalamic–pituitary–adrenal axis and abnormal cortisol secretion, which may be associated with lower EC capacity and stronger negative emotional response (Gotlib et al., 2026; Kuzminskaite et al., 2021).

Taken together, these empirical findings, derived from different types of stressors, consistently support a direct association between NLEs and IPs. The diathesis-stress model provides an important theoretical framework for understanding this relationship. According to this model, when individuals’ predispositional vulnerabilities interact with external stressful events, they become more susceptible to IPs (Ohashi et al., 2024). This risk may be particularly pronounced in adolescents, whose psychological regulatory capacities have not yet fully matured. Therefore, NLEs may not influence IPs solely through a direct effect; rather, they may operate through multiple underlying pathways that contribute to the emergence and development of internalizing symptoms in adolescents.

### 1.2 The mediating role of physical activity in the relationship between negative life events and internalizing problems

PA is widely regarded as an important protective factor for buffering stress and improving emotional well-being, and its beneficial effects have been supported by numerous studies. Research among adolescents has shown that regular PA can significantly reduce levels of depression and anxiety (Martinez et al., 2024). A meta-analysis further indicated that PA has been linked to lower depressive symptoms and a reduced likelihood of IPs in adolescents by regulating neurotransmitter systems, increasing the expression of dopamine, serotonin, and norepinephrine, and enhancing central nervous system plasticity (Wang T. et al., 2024).

Although PA has important physiological regulatory functions, high-stress situations are often associated with lower participation in PA among adolescents, which may correspond to lower availability of this protective behavioral resource. Studies have shown that NLEs, such as academic stress, are significantly negatively associated with participation in PA. Higher stress levels are often associated with maladaptive coping patterns, such as behavioral withdrawal, and with lower levels of PA. Although this deactivation of PA may temporarily help adolescents avoid stressors in the short term, prolonged withdrawal disrupts the exercise-mediated stress-buffering pathway (Lines et al., 2021; Ellis et al., 2015). In the absence of effective physiological release and psychological regulation strategies, adolescents may experience greater emotional dysregulation and become more vulnerable to IPs (McLaughlin and Hatzenbuehler, 2008; Stikkelbroek et al., 2016).

Taken together, PA may serve as an important protective factor in alleviating IPs, whereas NLEs, as a source of negative pressure, may weaken adolescents’ engagement in PA. This lack of PA may further exacerbate the depletion of cognitive resources caused by stress and ultimately contribute to the development of IPs.

### 1.3 The mediating role of emotional competence in the relationship between Negative Life Events and internalizing problems

EC is an important protective factor associated with adolescents’ IPs. A substantial body of research has shown that adolescents’ capacity to understand, manage, and use emotions is closely related to their adjustment when facing NLEs (Liu et al., 2022; Trevethan and Francis, 2025). Adolescents with stronger emotional competence may be better able to respond to stressful situations in a constructive manner, regulate negative emotional experiences, and avoid persistent maladaptive emotional responses (Daniel et al., 2020; McKone et al., 2024). A dual-factor model study further showed that EC is associated with fewer psychopathological symptoms and higher subjective well-being, making it a core element in the long-term maintenance of mental health (Guo et al., 2026). In the present study, EC is examined from an ability-oriented perspective and assessed as emotion regulation-related emotional competence using the Emotional Intelligence Scale. This perspective focuses on adolescents’ general capacity to perceive, manage, and use emotions.

NLEs, as environmental stressors, are often associated with symptoms such as depression and anxiety, particularly when accumulated stress exceeds adolescents’ psychological tolerance threshold. Higher exposure to NLEs may also co-occur with lower EC and greater difficulty in managing negative emotional experiences (Schönfeld et al., 2016). Previous research has indicated that NLEs are significantly associated with adolescents’ cognitive and emotional functioning, and that this relationship may involve psychological mediating mechanisms. When stress accumulates, adolescents may experience greater difficulty in regulating negative emotions and maintaining adaptive emotional functioning (Xie et al., 2025). Longitudinal trajectory studies have further suggested that stress-related emotional dysregulation is associated with later internalizing symptoms and reduced coping capacity when individuals encounter new stressors (Zhang et al., 2025).

Therefore, EC may represent a potential mediating factor in the relationship between NLEs and IPs. Examining this potential mediating mechanism is of practical significance for clarifying how environmental stress is associated with adolescents’ internal psychological difficulties and for developing targeted intervention strategies.

### 1.4 The chain mediating roles of physical activity and emotional competence

PA and EC may not operate in isolation in adolescents’ mental health; rather, they may be linked through interconnected behavioral and psychological pathways. As a modifiable behavioral factor, PA may provide adolescents with opportunities for behavioral activation, physiological release, peer interaction, self-efficacy, persistence, and frustration tolerance, all of which are relevant to emotional adjustment and self-regulatory functioning. Existing empirical studies have shown that higher levels of PA are associated with more adaptive emotion-related functioning. For example, Wang et al. found that university students with higher PA participation reported more frequent use of adaptive EC strategies, such as cognitive reappraisal (Wang et al., 2025a). Other studies have also suggested that PA is associated with positive psychological resources, such as self-efficacy and psychological resilience, which are closely related to emotional adjustment (Yan et al., 2024; Zhang et al., 2024). Through sustained behavioral engagement, PA may be associated with greater awareness of and perceived control over emotional states, making adolescents more likely to respond adaptively when confronted with stressful situations (Yang and Lu, 2025).

Accordingly, the present study proposes that PA and EC may constitute a closely linked chain mediation pathway. PA was theoretically positioned before EC because PA may create daily behavioral contexts in which adolescents can practice emotional awareness, self-control, persistence, frustration tolerance, and adaptive coping. This rationale is consistent with theoretical perspectives emphasizing the close connection between bodily activity, physiological regulation, and emotional regulation (Thayer and Lane, 2000; Bernstein, 2017). Therefore, PA may be associated with EC and may further be involved in adolescents’ adjustment to NLEs.

### 1.5 Research hypotheses

**Figure 1:**
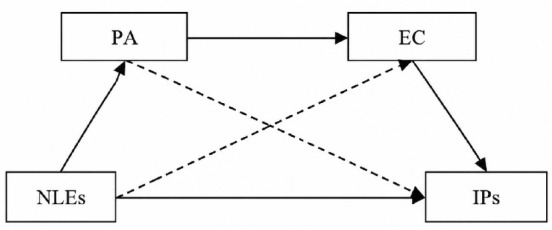
The theoretical model diagram. NLEs, Negative life events; IPs, internalizing problems; PA, physical activity; EC, emotional competence.

Taken together, existing studies remain somewhat fragmented in explaining the relationship between NLEs and adolescents’ IPs. Specifically, prior research has focused primarily on the independent mediating role of either PA or EC, with relatively little effort devoted to integrating these two factors within a unified theoretical framework and systematically examining whether they constitute a sequential chain mediation pathway. The lack of such an integrative perspective has, to some extent, constrained a deeper understanding of the internal mechanisms through which NLEs influence adolescents’ IPs (Figure 1).

Accordingly, drawing on the diathesis-stress model, the present study constructs and tests a chain mediation model incorporating PA and EC in order to clarify the pathways linking NLEs to IPs and to examine the mediating roles of PA and EC. In doing so, this study seeks to provide a theoretical basis for the development of more targeted and effective intervention strategies. Based on the above reasoning, the following hypotheses are proposed:

*H*_*1*:_ NLEs are significantly and positively associated with adolescents’ IPs.

*H_2_*: PA shows a significant potential indirect association in the relationship between NLEs and adolescents’ IPs.

*H_3_*: EC shows a significant potential indirect association in the relationship between NLEs and adolescents’ IPs.

*H_4_*: PA and EC jointly show a significant sequential indirect association between NLEs and adolescents’ IPs.

## 2 Methods

### 2.1 Participants

**Figure 2:**
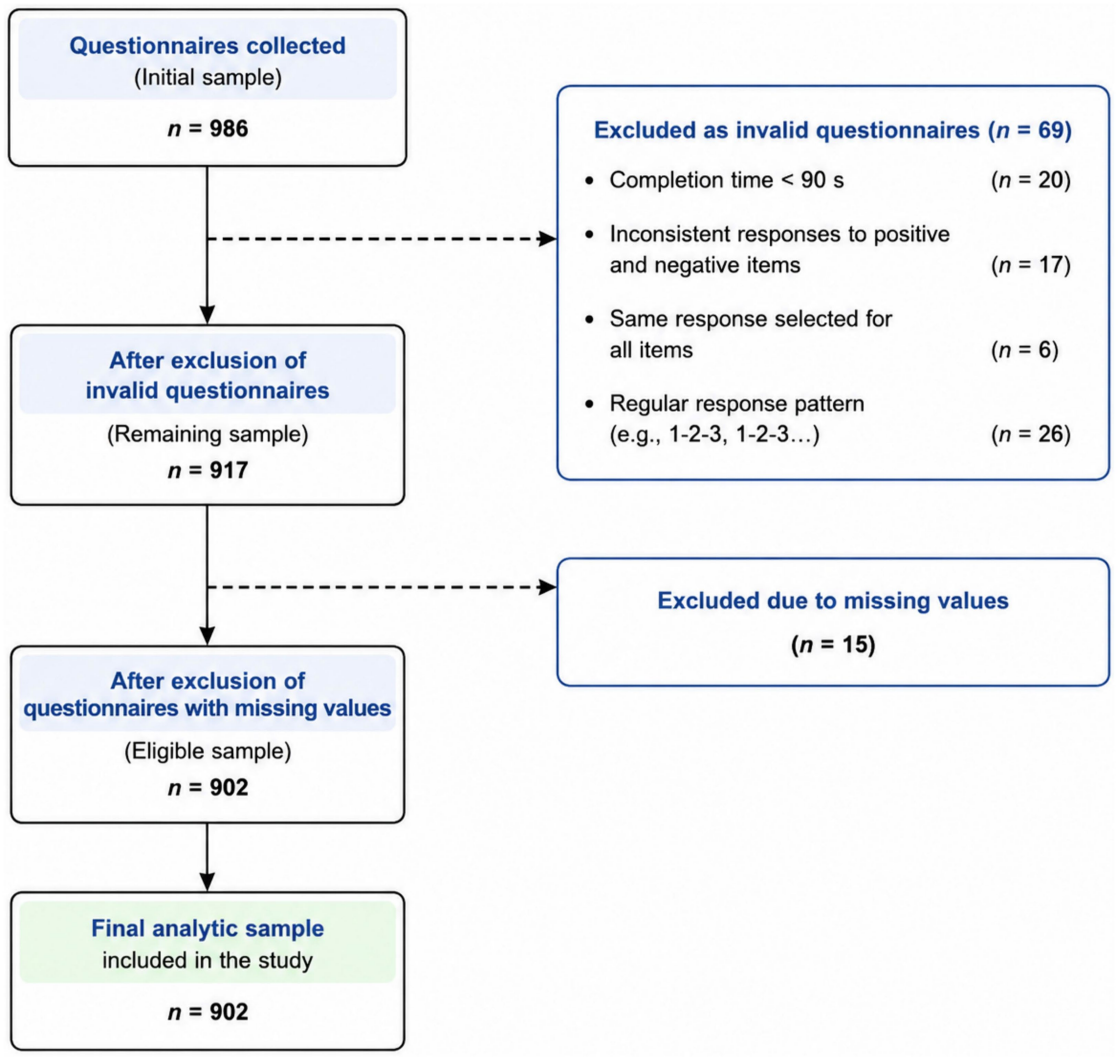
Steps in the screening process for research samples. Invalid questionnaires were identified based on: (1) completion time < 90 s, (2) inconsistent responses to positive and negative items, (3) same response selected for all items, and (4) regular response pattern (e.g., 1-2-3, 1-2-3…).

Participants in this study were recruited from five secondary schools in Henan Province, China, and a total of 902 adolescents were included in the final sample. A total of 986 questionnaires were distributed, of which 902 valid questionnaires were returned, yielding an effective response rate of 91.5%. The detailed screening procedure is presented in Figure 2. Among the 902 valid respondents, 438 (48.5%) were male and 464 (51.5%) were female. The mean age of the participants was 12.47 ± 2.21 years. Detailed demographic characteristics are shown in Table 1.

**Table 1:** Distribution of basic information on adolescents (*N* = 902).

Demographic variables		Number	Proportion%
Age	12.47 ± 2.21	902	100%
Sex	Male	438	48.5%
	Female	464	51.5%
Grade level	Middle school	429	47.5%
	High school	473	52.5%
Place of birth	Rural	382	42.3%
	Urban	520	57.7%
Only child (yes/no)	Yes	188	20.8%
	No	714	79.2%
Boarding student (yes/no)	Yes	621	68.8%
	No	281	31.2%

To ensure that the sample was consistent with the aims of the present study and to maintain data quality, clear inclusion and exclusion criteria were established in advance based on the research objectives and measurement requirements. The inclusion criteria were as follows: (1) full-time junior or senior high school students currently enrolled in school; (2) normal cognitive functioning and reading comprehension ability, with the capacity to understand and complete the self-report questionnaires independently and accurately; and (3) voluntary participation by the students themselves, with prior written informed consent obtained from their legal guardians. The exclusion criteria were as follows: (1) incomplete questionnaires or questionnaires showing obvious response patterns; (2) recent experience of physical trauma or the presence of medically advised exercise contraindications; (3) diagnosis of other neurodevelopmental disorders or neurological diseases; and (4) the presence of acute or chronic illnesses.

### 2.2 Procedure

This study was conducted in Henan Province, China, between March and April 2026. A combination of purposive sampling and convenience sampling was employed, and written informed consent was obtained from both the adolescents and their parents. The questionnaires were administered collectively in classroom settings, where trained research assistants provided standardized instructions and answered participants’ questions on site. All questionnaire administration and quality screening procedures were carried out by research assistants who had received prior training.

The questionnaires were completed by the participants themselves and collected immediately after completion. Following data collection, the researchers carefully checked each questionnaire for completeness, and any identified issues were promptly clarified with the participants in person. All questionnaires were distributed and retrieved face to face by the research team. Prior to the formal survey, permission to conduct the study was obtained through consultation with the local education authorities and the participating schools. Before completing the study questionnaires, all participants signed written informed consent forms and were informed of the purpose of the study, as well as its confidentiality and anonymity. This study was approved by the Biomedical Research Ethics Committee of Henan University, approval number: HUSOM2026-355.

### 2.3 Measures

#### 2.3.1 Negative life events

NLEs were assessed using the Adolescent Self-Rating Life Events Checklist (ASLEC) developed by Liu et al. (1997). The ASLEC was specifically designed for Chinese adolescents and consists of 26 items covering six domains: interpersonal relationships, academic stress, punishment, loss, health adaptation, and other events. The scale uses a 5-point Likert scoring format to assess the impact of adverse life events on adolescents. The total score is calculated by summing the scores of all items, with higher scores indicating greater psychological stress and more severe negative effects associated with experienced NLEs. The ASLEC has been validated in adolescent populations and has demonstrated good reliability and validity (Li et al., 2025; Tang et al., 2022; Zheng et al., 2025). In the present study, Cronbach’s *α* for the scale was 0.915.

#### 2.3.2 Internalizing problems

The Strengths and Difficulties Questionnaire (SDQ; Goodman, 1997) is a widely used screening instrument for assessing emotional and behavioral difficulties in adolescents (Goodman et al., 1998). The Chinese version has been translated and validated by Liu et al. (2013). Following previous studies, IPs were operationalized as the sum of the emotional symptoms and peer problems subscales of the SDQ (Zhu et al., 2025). The Chinese version of the SDQ has demonstrated good reliability and validity in adolescent samples and has been widely used in studies of internalizing problems. The scale uses a 3-point Likert format (0 = not true, 1 = somewhat true, 2 = certainly true). A total score was computed across relevant items, with higher scores indicating higher levels of IPs. In the present study, Cronbach’s *α* was 0.914.

#### 2.3.3 Physical activity

The Physical Activity Rating Scale (PARS-3) was developed by Liang (1994) and has been widely used in adolescent populations. The PARS-3 assesses three aspects of PA: intensity (“How intense is the physical activity you participate in?”), duration (“How long do you spend each time engaging in the above physical activity?”), and frequency (“How often do you participate in the above physical activity?”), each rated on a 5-point Likert scale. The total score was calculated as intensity × (duration − 1) × frequency, yielding a possible score range of 0 to 100, with higher scores indicating higher levels of PA participation. Previous studies have shown that the PARS-3 has good reliability and validity among adolescent populations (Nie et al., 2025). In the present study, Cronbach’s *α* for the scale was 0.788.

#### 2.3.4 Emotional competence

Emotional competence was assessed using the Emotional Intelligence Scale (Schutte et al., 1998; Shi and Wang, 2007), which has been widely validated in Chinese populations. The scale includes 33 items across four dimensions: emotional perception, self-emotion management, others’ emotion management, and emotion utilization. Because the EIS captures broader emotional competence rather than specific emotion regulation strategies, it was used as a proxy measure of emotional competence in this study. Higher scores indicate stronger emotional competence. Cronbach’s α in the present sample was 0.947, indicating excellent internal consistency, which is consistent with previous validation findings in Chinese college student samples (Wang et al., 2026).

### 2.4 Data analysis

All statistical analyses were performed using SPSS 27.0. Harman’s single-factor test was conducted to examine potential common method bias. Descriptive statistics and Pearson correlation analyses were conducted to examine the distributions and associations among variables. Multiple linear regression analyses were performed to test the main effects. Mediation analyses were conducted using PROCESS Model 6 (SPSS macro, version 3.3) with 5,000 bootstrap resamples and 95% confidence intervals. All mediation analyses controlled for sex, age, grade level, place of birth, only-child status, and boarding status. Standardized coefficients (*β*) were reported in the multiple linear regression analyses, whereas unstandardized coefficients (b) were used for mediation analyses.

## 3 Results

### 3.1 Common method bias test

Because all variables were assessed through self-report measures, Harman’s single-factor test was used to evaluate the potential influence of common method bias. The results showed that 14 common factors with eigenvalues greater than 1 were extracted through principal component analysis, accounting for 59.96% of the total variance. The unrotated variance explained by the first factor was 23.27%, which was well below the threshold of 40%, indicating that no serious common method bias was present in the data.

### 3.2 Descriptive statistics and correlation analysis

Table 2 presents the means, standard deviations, and correlations of NLEs, IPs, PA, and EC. The mean score for NLEs (43.99 ± 11.71) indicated a relatively high level of exposure to NLEs. The mean score for IPs (11.22 ± 5.13) suggested a moderate level of IPs among the adolescents. The mean PA score (22.74 ± 22.33) indicated that adolescents’ PA level was slightly below the moderate range.

**Table 2:** Means, standard deviations and correlations among all variables (*N* = 902).

Variable	*M*	*SD*	1	2	3	4
1. Negative life events	43.99	11.71	**1**			
2. Internalizing problems	11.22	5.13	0.449^**^	**1**		
3. Physical activity	22.74	22.33	−0.356^**^	−0.357^**^	**1**	
4. Emotional competence	120.38	26.177	−0.358^**^	−0.375^**^	0.401^**^	**1**

**p < 0.01.

The correlation analyses showed that the independent variable NLEs was significantly and positively correlated with the dependent variable IPs (*r* = 0.45, *p* < 0.01). The mediating variables PA (*r* = −0.357, *p* < 0.01) and EC (*r* = −0.375, *p* < 0.01) were both significantly and negatively correlated with IPs. In addition, NLEs were negatively correlated with both PA (*r* = −0.356, *p* < 0.01) and EC (*r* = −0.358, *p* < 0.01). A significant positive correlation was observed between the two mediators, PA and EC (*r* = 0.401, *p* < 0.01) (see Table 2).

### 3.3 Regression analysis

To examine whether NLEs, PA, and EC were associated with IPs, hierarchical multiple regression analyses were conducted. The results are presented in Table 3. Prior to the regression analyses, multicollinearity diagnostics were performed for all predictor variables. The results showed that the variance inflation factor (VIF) values ranged from 1.222 to 1.272, well below the threshold of 10, indicating that there was no serious multicollinearity in the data and that the variables were suitable for subsequent regression and mediation analyses.

**Table 3:** Results of regression analysis.

Variable		Dependent variable: IPs								Dependent variable:PA		Dependent variable:EC	
		Model 1		Model 2		Model 3		Model 4		Model 5		Model 6	
		β	*t*	β	*t*	β	*t*	β	*t*	β	*t*	β	*t*
Constant		13.09	8.07	3.00	1.87	4.09	2.60	9.32	5.29	21.65	3.06	135.63	16.47
Control variable	Sex	0.06	1.70	0.04	1.47	0.28	0.94	0.04	1.43	−0.08*	−2.49	0.07*	2.30
	Age	−0.01	−0.27	−0.01	−0.02	0.01	0.26	0.01	0.22	0.04	1.26	0.01	−0.24
	Grade level	−0.09**	−2.74	−0.06*	−2.01	−0.05	−1.54	−0.04	−1.47	0.07*	2.28	0.02	0.50
	Place of birth	−0.07*	−2.20	−0.05	−1.56	−0.03	−1.15	−0.04	−1.41	0.06	1.94	−0.04	−1.15
	Only child or not	−0.04	−1.23	−0.02	−0.60	0.03	1.00	0.03	0.99	0.23*	2.38	−0.01	−0.11
	Boarding or not	0.08*	2.28	0.08*	2.75	0.08*	2.58	0.07*	2.46	−0.03	−1.06	−0.03	−0.83
Independent variable	NLEs	-	-	0.44***	14.79	0.37***	11.85	0.32***	10.16	−0.33***	−11.09	−0.25***	−7.78
Mediating variable	PA	-	-	-	-	−0.22***	−6.80	−0.16***	−4.69	-	-	0.32***	9.77
	EC	-	-	-	-	-	-	−0.20***	−6.14	-	-	-	-
Model fit	*R*	0.16		0.47		0.51		0.54		0.45		0.47	
	*R^2^*	0.03		0.22		0.26		0.29		0.20		0.22	
	Adjusted *R^2^*	0.02		0.21		0.25		0.28		0.20		0.21	
	*F*	4.00***		35.53***		38.45***		39.77***		31.52***		31.56***	

NLEs, Negative life events; IPs, Internalizing problems; PA, Physical activity; EC, Emotional Competence. *p < 0.05, **p < 0.01, ***p < 0.001.

First, in Model 1, demographic variables, including gender, age, grade, place of birth, and only-child status, were entered as control variables. Next, NLEs were added in Model 2, and the results showed that NLEs significantly predicted an increase in IPs (*β* = 0.44, *p* < 0.001), thereby supporting H_1_. In addition, the results of Model 3 indicated that PA significantly predicted a decrease in IPs (*β* = −0.22, *p* < 0.001). In Model 4, EC was further found to significantly predict a decrease in IPs (*β* = −0.20, *p* < 0.001).

Moreover, as shown in Model 5, NLEs had a significant negative effect on PA (*β* = −0.33, *p* < 0.001). Finally, the results of Model 6 indicated that PA significantly and positively predicted EC (*β* = 0.32, *p* < 0.001). Overall, the hierarchical regression analyses clearly demonstrated the interrelationships among NLEs, PA, and EC, with each variable showing a significant effect in predicting IPs. Standardized coefficients (β) were reported for regression analyses, whereas unstandardized coefficients (b) were used for mediation analyses.

### 3.4 Mediation analysis

**Figure 3:**
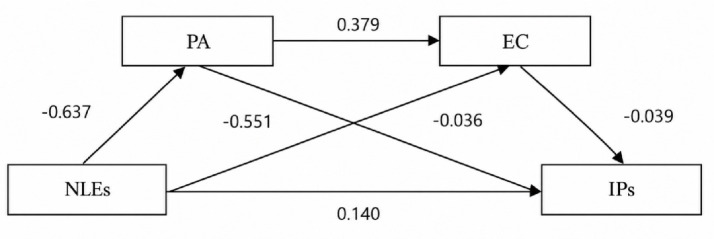
Serial mediation model with unstandardized path coefficients. NLEs, Negative life events; IPs, internalizing problems; PA, physical activity; EC, emotional competence.

The results of the chain mediation analysis are presented in Table 4 and Figure 3. NLEs had a significant direct effect on IPs (*b* = 0.140, 95%CI [0.113, 0.167]), accounting for 72.33% of the total effect. In addition, PA and EC jointly exerted a significant indirect effect on the relationship between NLEs and IPs (*b* = 0.053, 95%CI [0.040, 0.067]), which accounted for 27.66% of the total effect. These findings indicate the presence of a chain mediation pathway, whereby PA and EC jointly mediate the impact of NLEs on adolescents’ IPs.

**Table 4:** Mediating effects and effect sizes.

Path	Effect	*SE*	Bootstrap 95%CI		Percentage of total effect
			Lower	Upper	
Total effect	0.193	0.013	0.167	0.218	-
Direct Effect	0.140	0.014	0.113	0.167	72.33%
Total indirect effects	0.053	0.007	0.040	0.067	27.66%
Ind1: NLEs →PA → IPs	0.023	0.006	0.012	0.034	11.82%
Ind2: NLEs →EC → IPs	0.021	0.005	0.013	0.031	10.99%
Ind3: NLEs →PA → EC → IPs	0.009	0.002	0.006	0.014	4.82%

Specifically, the mediation analysis for Path Ind1 showed that PA significantly mediated the association between NLEs and adolescents’ IPs (*b* = 0.023, 95%CI [0.012, 0.034]), accounting for 11.82% of the total effect. This finding supports H_2_ and suggests that PA serves as a mediator in the relationship between NLEs and adolescents’ IPs. The indirect effect analysis further showed that EC also significantly mediated the relationship between NLEs and adolescents’ IPs (*b* = 0.021, 95%CI [0.013, 0.031]), accounting for 10.99% of the total effect. This result supports H_3_, indicating that EC plays a mediating role between NLEs and adolescents’ IPs. Moreover, the joint mediating effect of PA and EC in the association between NLEs and adolescents’ IPs was also significant (*b* = 0.009, 95%CI [0.006, 0.014]), accounting for 4.82% of the total effect. This finding provides support for H_4_.

## 4 Discussion

### 4.1 Negative life events as contextual stressors associated with adolescents’ internalizing problems

The present study found a significant positive association between NLEs and adolescents’ IPs. Rather than indicating a causal pathway, this finding suggests that adolescents who reported more frequent or intense negative life experiences also tended to report higher levels of internalizing difficulties. This pattern is consistent with the diathesis-stress model, which emphasizes that adolescents’ psychological adjustment is shaped by the interaction between environmental stressors and developing regulatory capacities. During adolescence, cognitive, emotional, and social systems are still undergoing rapid development; therefore, repeated exposure to stressful experiences may be associated with greater difficulty in maintaining psychological balance and adaptive functioning. This association is particularly meaningful in educational contexts. For junior and senior high school students, many NLEs are embedded in daily school life, including academic pressure, examination stress, peer conflict, teacher-student relationships, and expectations for educational advancement. Academic stress, in particular, represents one of the most persistent and developmentally salient stressors for adolescents (Chen and Guo, 2026). Previous evidence has shown that academic stress and the timing of the academic year are closely associated with adolescent mental health outcomes (Steare et al., 2023). These findings suggest that schools are not only settings in which stress may accumulate, but also important environments where adolescents’ behavioral resources, emotional competence, and psychological support can be shaped.

From a developmental adaptation perspective, the positive association between NLEs and IPs may reflect an imbalance between stress exposure and available coping resources. This suggests that adolescent IPs should not be understood only as individual emotional difficulties, but also as adjustment difficulties embedded in broader developmental and educational environments. Therefore, schools and families may need to identify adolescents exposed to frequent negative life experiences at an earlier stage and provide more concrete support. For example, schools could strengthen routine mental health screening, provide counseling services for students experiencing academic or interpersonal stress, and create supportive classroom and peer environments. Families could also reduce excessive achievement pressure, improve parent-adolescent communication, and provide stable emotional support. These efforts may help reduce the psychological burden associated with NLEs and provide a foundation for the behavioral and emotional support discussed in the following sections.

### 4.2 Physical activity as a behavioral resource in the association between negative life events and internalizing problems

The present findings suggest that PA may be involved in the association between NLEs and adolescents’ IPs. Specifically, adolescents who reported more NLEs tended to report lower levels of PA, and lower PA was associated with higher levels of IPs. This pattern is consistent with the stress-induced exercise suppression hypothesis, which suggests that acute or chronic stress may be accompanied by reduced behavioral motivation and lower participation in PA (Stults-Kolehmainen and Sinha, 2014). Previous studies have similarly shown that adolescents exposed to higher levels of academic stress or interpersonal conflict often report lower levels of PA (Haag et al., 2023; Hartson et al., 2023). From this perspective, PA may be understood not only as a health behavior, but also as a behavioral resource that is closely related to adolescents’ capacity to maintain adjustment under stress.

This finding is particularly relevant in school settings. For adolescents, opportunities for PA are strongly shaped by school schedules, physical education arrangements, academic workload, peer relationships, and access to extracurricular activities. When academic pressure increases or school routines become dominated by examination preparation, students may have fewer opportunities or less motivation to engage in regular PA. Reduced PA may further limit adolescents’ access to behavioral activation, physiological release, peer interaction, and positive self-efficacy experiences, all of which are relevant to psychological adjustment (Dollard et al., 2013). In this sense, PA may provide an important behavioral context through which adolescents manage stress and maintain emotional balance.

At the same time, PA should not be interpreted as a single or sufficient solution for adolescents’ IPs. Rather, the present findings suggest that PA may be one modifiable behavioral factor within a broader system of adolescent wellbeing. Previous studies among adolescents have also reported that higher PA is associated with lower levels of internalizing or emotional difficulties (Bartels et al., 2012; Wu et al., 2025). For school-based practice, this implies that structured physical education programs, after-school sports activities, and team-based exercise programs may be useful ways to increase adolescents’ access to regular PA, especially for students experiencing academic or interpersonal stress. Families may also support adolescents by encouraging regular movement, reducing excessive academic pressure, and helping them maintain balanced daily routines. Such behavioral support may provide a practical foundation for broader psychological interventions targeting adolescents exposed to NLEs.

### 4.3 Emotional competence as a psychological resource

The present findings suggest that EC may be involved in the association between NLEs and adolescents’ IPs. Adolescents who reported more NLEs tended to report lower EC, and lower EC was associated with higher levels of IPs. This pattern is consistent with developmental perspectives emphasizing that adolescents’ ability to perceive, manage, and use emotions is closely related to their psychological adjustment under stress.

From the perspective of emotion regulation and developmental adaptation, cumulative negative experiences may be associated with greater demands on adolescents’ cognitive and emotional resources. When adolescents encounter repeated academic, interpersonal, or family-related stressors, they may experience greater difficulty in organizing emotional experiences, maintaining emotional balance, and responding adaptively to stressful situations. Previous research based on the process model of emotion regulation suggests that stress may place demands on cognitive control and emotional processing systems, which are central to adolescents’ adjustment (Ochsner and Gross, 2005). Similarly, studies have shown that stress-related emotional dysregulation is associated with higher levels of internalizing symptoms and weaker coping capacity (Stikkelbroek et al., 2016; Guassi Moreira et al., 2022; Hossein et al., 2023).

EC may therefore function as an important psychological resource in adolescents’ adaptation to NLEs. Adolescents with stronger emotional competence may be better able to recognize negative emotions, understand their emotional responses, and manage distress in ways that support adaptive functioning. This interpretation is consistent with previous research showing that emotional competence and regulation-related abilities are associated with fewer internalizing symptoms and better psychological adjustment among adolescents (Compas et al., 2017; McLaughlin et al., 2011; Hsieh and Stright, 2012). Rather than emphasizing a single regulation strategy, the present findings highlight the broader role of emotional competence in helping adolescents maintain psychological stability when facing stressful experiences. Taken together, EC may represent a meaningful psychological resource associated with adolescents’ adjustment to NLEs. This finding also has implications for educational wellbeing practice. Schools may strengthen adolescents’ emotional competence through emotion-related curricula, mindfulness-based activities, and accessible counseling services, while families may provide emotional support and encourage open communication, particularly for adolescents exposed to frequent NLEs.

### 4.4 The chain mediating roles of physical activity and emotional competence

The present study further examined whether PA and EC may jointly be involved in the association between NLEs and adolescents’ IPs. The findings were consistent with the hypothesized statistical model, suggesting that PA and EC may form a behavioral-emotional pathway through which adolescents’ exposure to NLEs is associated with internalizing difficulties. From a developmental adaptation perspective, PA may provide adolescents with opportunities for behavioral activation, physiological release, self-efficacy, and peer interaction, all of which are relevant to emotional adjustment and self-regulatory functioning (Singh et al., 2023). When adolescents exposed to more NLEs report lower PA, they may have fewer opportunities to access these behavioral resources. Reduced behavioral engagement may in turn be associated with lower EC, as adolescents may have fewer daily contexts in which they can practice emotional awareness, persistence, frustration tolerance, and adaptive coping. This interpretation is consistent with views emphasizing the close connection between bodily activity, emotional regulation, and psychological adjustment (Bernstein, 2017; Thayer and Lane, 2000). However, the ordering of PA and EC should be interpreted as a theoretically specified statistical sequence rather than a confirmed developmental process. Given the cross-sectional design, the directionality among these variables cannot be determined. Alternative models are equally plausible. For instance, adolescents with higher levels of internalizing problems may be less likely to engage in physical activity due to reduced motivation and energy, and may also report poorer emotional functioning. In addition, PA, EC, and IPs may be reciprocally related, forming bidirectional or mutually reinforcing associations over time. Therefore, the proposed sequential pathway should not be interpreted as a developmental trajectory. Longitudinal designs, particularly cross-lagged panel models or intervention-based studies, are required to validate the temporal ordering and causal direction of the observed associations before such a sequence can be considered a developmental mechanism linking NLEs to IPs.

This pathway is also meaningful in educational contexts. Schools may simultaneously shape adolescents’ stress exposure, opportunities for PA, and development of emotional competence through academic workload, peer relationships, physical education, extracurricular activities, and psychological support services. Therefore, adolescent IPs should not be understood only as individual psychological difficulties, but also as adjustment difficulties embedded in broader educational environments. However, this sequential pathway should be interpreted cautiously. Although statistically significant, the PA–EC sequential indirect effect accounted for only 4.82% of the total association between NLEs and IPs, indicating a relatively small effect size. This finding suggests that PA and EC may constitute a specific but limited behavioral-emotional pathway, rather than the dominant mechanism linking NLEs to IPs. In practice, schools and families may consider integrated support that combines structured PA opportunities with emotion-related education and accessible psychological services, rather than relying on any single intervention approach.

## 5 Limitations and future directions

Several limitations of the present study should be acknowledged. First, EC was examined from an ability-oriented perspective and assessed using the EIS. Although this scale captures emotional competence relevant to emotional competence, it does not directly measure specific emotion regulation strategies. Therefore, the findings should be interpreted as reflecting emotion regulation-related emotional competence. Future studies could use more specialized instruments, such as the Emotion Regulation Questionnaire, Difficulties in Emotion Regulation Scale, or Cognitive Emotion Regulation Questionnaire, to assess specific regulation strategies more directly.

Second, all variables were assessed using single-occasion self-report questionnaires, which may introduce common method variance and systematic response tendencies, such as social desirability and recall-related reporting errors. Although Harman’s single-factor test did not suggest serious common method bias, this test is insufficient as a standalone diagnostic and cannot fully exclude shared method effects. Thus, the observed associations and indirect effects may have been inflated to some extent by the common measurement source. This concern is particularly relevant to PA, which was assessed by self-report. Future studies should adopt multi-method designs by integrating objective PA measures, such as accelerometers or wearable devices, with multi-informant reports from parents, teachers, or peers.

Third, although demographic covariates were included, potentially important confounders were not assessed. Socioeconomic status may condition exposure to NLEs, access to PA, and vulnerability to IPs, whereas family functioning and social support may buffer stress effects and support EC. Sleep quality and academic performance may also covary with PA, regulatory capacity, and internalizing symptoms. Accordingly, omitted-variable bias and residual confounding cannot be excluded. The identified mediation pathways should therefore be interpreted as partial statistical associations rather than a comprehensive explanatory model of the relationship between NLEs and IPs. Future research should incorporate broader individual, family, school, and social-contextual covariates.

Finally, this study adopted a cross-sectional design. Although such a design can reveal associations among variables, it cannot establish their temporal order or causal direction. In particular, the present design cannot determine whether PA precedes EC, whether EC influences adolescents’ participation in PA, or whether the two constructs are reciprocally related. Future studies should use longitudinal tracking, cross-lagged analyses, and intervention designs to further examine the dynamic associations among NLEs, PA, EC, and IPs, thereby providing stronger empirical support for adolescent mental health interventions.

## 6 Conclusion

The present study yielded the following conclusions. NLEs, PA, EC, and adolescents’ IPs were all significantly correlated with one another. Specifically, PA and EC each played a significant mediating role in the relationship between NLEs and adolescents’ IPs, and the relevant pathways were all characterized by negative predictive associations. In addition, NLEs could indirectly influence adolescents’ IPs through the sequential chain mediation pathway involving PA and EC. Thus, NLEs were directly associated with adolescents’ IPs in the statistical model, but also showed significant indirect associations through the independent mediating roles of PA and EC as well as their joint chain mediating effect.

## Data Availability

The raw data supporting the conclusions of this article will be made available by the authors, without undue reservation.
